# Alterations in the Fecal Microbiome and Metabolome of Horses with Antimicrobial-Associated Diarrhea Compared to Antibiotic-Treated and Non-Treated Healthy Case Controls

**DOI:** 10.3390/ani11061807

**Published:** 2021-06-17

**Authors:** Carolyn Arnold, Rachel Pilla, Keith Chaffin, Jonathan Lidbury, Joerg Steiner, Jan Suchodolski

**Affiliations:** 1 Department of Large Animal Clinical Sciences, College of Veterinary Medicine & Biomedical Sciences, Texas A&M University, College Station, TX 77843, USA; KChaffin@cvm.tamu.edu; 2 Department of Small Animal Clinical Sciences, College of Veterinary Medicine & Biomedical Sciences, Texas A&M University, College Station, TX 77843, USA; RPilla@cvm.tamu.edu (R.P.); JLidbury@cvm.tamu.edu (J.L.); JSteiner@cvm.tamu.edu (J.S.); JSuchodolski@cvm.tamu.edu (J.S.)

**Keywords:** colitis, antimicrobial-associated diarrhea, microbiota, metabolome

## Abstract

**Simple Summary:**

Antimicrobials reduced the diversity and altered the bacterial community composition of the fecal microbiome of horses. Horses that develop antimicrobial-associated diarrhea (AAD) show greater derangements in their bacterial community composition compared to antibiotic (ABX) and non-antibiotic (CON) control horses. Horses with AAD have altered metabolic profiles compared to ABX and CON horses.

**Abstract:**

Diarrhea is an adverse effect of antimicrobial therapy in horses. This matched, case-controlled study compared the fecal microbiome and metabolome of horses on antibiotics that developed diarrhea (AAD, *n* = 17) to those that did not develop diarrhea (ABX, *n* = 15) and to a control population not exposed to antibiotics (CON, *n* = 31). Fecal samples were collected from horses that were matched for diet and antimicrobial agent (including dose, route, and duration of therapy). Illumina sequencing of 16S rRNA genes was performed, and QIIME 2.0 was used to generate alpha and beta diversity metrics. Untargeted metabolomics using GC-MS platforms was performed and analyzed using Metaboanalyst 5.0. Microbiome composition was significantly different in AAD compared to CON (ANOSIM, R = 0.568, *p* = 0.001) but not to ABX (ANOSIM, R = 0.121, *p* = 0.0012). AAD and ABX horses had significantly decreased richness and evenness compared to CON horses (*p* < 0.05). Horses on antimicrobials (AAD and ABX) had significant changes in 14 phyla compared to CON horses. Only Verrucomicrobia distinguished AAD from ABX and CON horses (q = 0.0005). Metabolite profiles of horses with AAD clustered separately from ABX and CON horses. Seven metabolites were found to be significantly different between groups (*p* < 0.05): L-tyrosine, kynurenic acid, xanthurenic acid, 5-hydroxyindole-3-acetic acid, docosahexaenoic acid ethyl ester, daidzein, and N-acetyltyramine. Metabolite profiles of horses on antimicrobials, especially those with AAD, are altered compared to CON horses.

## 1. Introduction

Antimicrobial-associated diarrhea (ADD) is a common adverse effect of antibiotic use in horses [1]. Although all antimicrobials have potential to cause ADD, some antibiotic agents have increased risk due to biliary excretion, enterohepatic recycling, or the concentration of the drug in the intestinal lumen secondary to low oral absorption [2,3]. To date, tetracyclines [4,5,6], macrolides [7,8,9], cephalosporins [10,11], fluoroquinolones [5], trimethoprim-sulphonamides [6,12,13], chloramphenicol [14], β-lactams [10,13,15], and metronidazole [13,15,16] have been reported to cause AAD in horses. 

Because AAD is not well defined in the veterinary literature regarding stool character, frequency, temporal association to antibiotic administration, or degree of resulting illness, the true incidence in the equine population is difficult to define. In addition, AAD appears to have a disproportionate impact on horses residing in a hospital setting. A multicenter study found that AAD occurred in <1% of weanling to adult horses living on farms with an associated mortality rate of 19% [5]. In veterinary referral centers, AAD has been reported to range from 22 to 94% in adult horses with mortality rates ranging from 15 to 50% [4,5,8]. One retrospective study found that horses with AAD were 4.5 times more likely to die compared to horses with other types of colitis [17]. These factors indicate an urgent need to better understand why AAD develops, to identify individuals at risk, and to improve the morbidity and survival rates of horses with this disease. 

Recent advances in molecular technologies, such as metagenomics and metabolomics, may provide insight into equine AAD. Next-generation sequencing techniques have enabled the identification of thousands of bacterial species previously unrecognized by traditional culture-based techniques. Collectively known as the microbiota, the bacteria that reside in the gut play a functional role in critical metabolic processes, such as nutrient digestion and absorption, the production of short chain fatty acids and bile acids, the biosynthesis of vitamins and amino acids, the regulation of the inflammatory environment of the gut, and the modulation of the immune system against pathogens [11]. Changes in the bacterial community of the gut that occur in association with states of disease, a phenomenon known as dysbiosis, are accompanied by alterations in these physiologically important metabolic processes [11]. Metabolomic analysis may be used to identify the specific pathways and metabolites altered by AAD. In both humans and companion animal species, dysbiosis of the gut microbiome has been strongly associated with the presence of colitis. In horses, studies employing metagenomic methods have indicated that colitis [18,19,20] and antimicrobial agents reduce microbiome’s diversity [10,16,21,22]; however, there are few studies to date that have characterized either the microbiome or metabolome of horses with AAD [23,24]. It is unknown if horses with AAD develop greater dysbiosis or metabolic alterations than horses on antimicrobial agents that maintain normal fecal character and health status. Therefore, the purpose of this study was to compare the fecal microbiome and metabolome of three populations of horses: those with diarrhea as a result of antibiotic administration (AAD), those on antibiotics that did not develop diarrhea (ABX), and healthy horses not on antibiotics (CON).

## 2. Materials and Methods

### 2.1. Subjects

The inclusion criteria for all study participants were a horse of at least one year of age, of any breed or sex, and with no prior history of gastrointestinal disease. Fecal samples from AAD and ABX horses were collected at the Texas A&M Veterinary Medical Teaching Hospital (College Station, TX, USA). These horses were prescribed antimicrobials as prophylaxis before elective surgery (excluding surgery of the gastrointestinal tract including colic) or to treat a suspected or confirmed infection. AAD horses were defined as those that developed colitis during the course of antimicrobial therapy and were classified by the clinician as having colitis secondary to antibiotic administration. Horses with AAD developed diarrhea (increased water content in feces, feces that was no longer formed) in combination with lethargy, fever, and inappetence. All horses with AAD required treatment (discontinuation of antimicrobial agent and therapy to address hypovolemia, endotoxemia, electrolyte deficiencies, etc.). ABX horses were defined as those that maintained normal health status and fecal character throughout the course of antimicrobial therapy. Fecal samples from non-hospitalized control horses (CON) were collected as part of an earlier study [23] and had no history of antibiotic administration, non-steroidal anti-inflammatory (NSAID) use, or gastrointestinal disease for the last 6 months. AAD and ABX horses were matched by the specific antimicrobial and the duration of antibiotic therapy on which diarrhea developed in the AAD horse (less than or greater than 5 days). All horses were matched by diet according to the following categories: A, forage only (hay and/or pasture); B, forage plus low fiber concentrate (5–7% maximum crude fiber) fed at ≤0.5% of body weight in kg/day; C, forage plus medium fiber concentrate (10–15% maximum crude fiber) fed at ≤0.5% of body weight in kg/day; D, forage plus high fiber concentrate (18–33% maximum crude fiber) fed at ≤ 0.5% of body weight in kg/day; E, forage plus medium fiber concentrate (10–15% maximum crude fiber) fed at 1–2% of body weight in kg/day [23]. Fecal samples were collected after natural elimination and stored at −80 °C until processed in the lab. 

### 2.2. Microbiome 

#### 2.2.1. Sample Preparation

DNA was extracted from fecal samples using the PowerSoil DNA Isolation Kit (MO BIO, Carlsbad, CA, USA) following the manufacturer’s instructions. Sequencing of the V4 region of the 16S rRNA gene using primers 515F (5′-GTGYCAGCMGCCGCGGTAA) [24] to 806RB (5′-GGACTACNVGGGTWTCTAAT) [25,26] was performed at MR DNA (www.mrdnalab.com, Shallowater, TX, USA) on an Illumina MiSeq platform (Illumina Inc., San Diego, CA). Quantitative Insights into Microbial Ecology (QIIME 2) v2018.6 [27] was used for analysis of the sequences. Raw sequence data were uploaded to NCBI Sequence Read Archive under project PRJNA728793. The sequences were demultiplexed and the ASV table was created using DADA 2. Prior to downstream analysis, sequences assigned to mitochondria, chloroplasts, cyanobacteria, or low abundance ASVs containing less than 0.01% of the total reads were excluded from further analysis. All samples were rarefied to even sequencing depth of 88,730,000 sequences per sample. Alpha diversity was measured with the Chao1 (richness), Shannon diversity, and observed ASVs metrics within QIIME2. Beta diversity was evaluated with the phylogeny-based UniFrac [28] distance matrices and visualized using principal coordinate analysis (PCoA) plots, generated within QIIME2. 

#### 2.2.2. Statistical Analysis of Microbiome Data

As data followed a non-normal distribution according to a Shapiro–Wilk test (JMP Pro 14, SAS, Marlow, Buckinghamshire, UK), non-parametric measures were used throughout the study. Statistical analysis of alpha diversity indices (Chao 1, amplicon sequence variants (ASVs), and Shannon) was performed using a Kruskal–Wallis test with a Dunn’s multiple comparison post-test in the software package PRISM (PRISM 8, GraphPad Software Inc., San Diego, CA, USA). An analysis of similarity test (ANOSIM) within the PRIMER 6 (PRIMER-E Ltd. Luton, UK) software package was performed on the beta diversity distance matrices to assess the significance of the differences in the bacterial community composition. R values that result from ANOSIM testing can be described as follows: 0.75 < R < 1, highly different; 0.5 < R < 0.75, different; 0.25 < R < 0.5, different with some overlap; 0.1 < R < 0.25, similar with some differences or high overlap; R < 0.1, similar.

Analysis of the bacterial taxa in the fecal samples was evaluated using a Kruskal–Wallis test (PRISM 8, GraphPad Software Inc., San Diego, CA, USA) followed by a Dunn’s multiple comparison post-test. 

Linear discriminant analysis effect size (LEfSe) using the web-based program Calypso v8.62 (http://cgenome.net/wiki/index.php/Calypso, accessed on 21 February 2021) was performed to analyze the abundance of bacterial taxa and their associations with group (AAD, ABX, and CON). A cut-off threshold of 3.5 was set for significance. 

### 2.3. Metabolome

#### 2.3.1. Sample Preparation

An amount of 500 mg of feces was aliquoted into a 2 mL tube, lyophilized overnight, and vortexed with a 5 mm stainless steel bead (Quiagen, Germantown, MD, USA) for 5 min. Samples were then extracted using a methanol:chloroform:water-based extraction method. Briefly, 800 uL of ice-cold methanol:chloroform (1:1, v:v) was added to samples in a bead-based lysis tube (Bertin, Rockville, MD, USA). Samples were homogenized for 30 s on a Precyllys 24 (Bertin) at a speed of 6000. The supernatant was collected and samples were homogenized a second time with 800 uL of ice methanol:chloroform. An amount of 600 uL of ice-cold water was added to the combined extract, vortexed, and centrifuged to separate the phases. The upper aqueous layer was passed through a 0.2 um nylon filter (Merck Millipore, Burlington, MA, USA). An amount of 500 uL of the filtered aqueous phase was then passed through a 3 kDa cutoff column (Thermo Scientific, Waltham, MA, USA), and the flow through was collected for analysis. 

#### 2.3.2. Metabolomic Analysis

Untargeted liquid chromatography high-resolution accurate mass spectrometry (LC-HRAM) analysis was performed on a Q Exactive Plus orbitrap mass spectrometer (Thermo Scientific) coupled to a binary pump HPLC (UltiMate 3000, Thermo Scientific). Full MS spectra were obtained at 70,000 resolution (200 m/z) with a scan range of 50–750 m/z. Full MS followed by ddMS2 scans were obtained at 35,000 resolution (MS1) and 17,500 resolution (MS2) with a 1.5 m/z isolation window and a stepped NCE (20, 40, 60). Samples were maintained at 4 °C before injection. The injection volume was 10 µL. Chromatographic separation was achieved on a Synergi Fusion 4µm, 150 mm × 2 mm reverse phase column (Phenomenex, Torrance, CA, USA) maintained at 30 °C using a solvent gradient method. Solvent A was water (0.1% formic acid). Solvent B was methanol (0.1% formic acid). The gradient method used was 0–5 min (10% B to 40% B), 5–7 min (40% B to 95% B), 7–9 min (95% B), 9–9.1 min (95% B to 10% B), 9.1–13 min (10% B). The flow rate was 0.4 mL min^−1^. Sample acquisition was performed Xcalibur (Thermo Scientific). Data analysis was performed with Compound Discoverer 3.1 (Thermo Scientific).

#### 2.3.3. Statistical Analysis of Metabolomic Data

MetaboAnalyst 5.0 (Xia Lab, McGill University, Canada) was used to analyze metabolomics data. Unnamed peaks and peaks related to antibiotics were removed prior to analysis. The peak intensity data table contained peak heights normalized against the average total peak sums. Data were not filtered but were log transformed and subjected to Pareto scaling. Metabolites were cross referenced using their compound number to the KEGG database (www.genome.jp/kegg/pathway, accessed on 22 January 2021) in order to identify their metabolic pathways, such as metabolism, cellular processes, and others.

A principal component analysis plot (PCA) was used to display the metabolomic composition of each sample and visualize separation between groups. A heat map was used to display the hierarchical clustering of metabolites by concentration in each individual horse and group. An ANOVA followed by a Tukey’s post-test was used to determine which metabolites were significantly different between groups. Random forest modelling was performed to predict which metabolites were associated with AAD, ABX, or CON horses. Metabolomic data have been submitted to metabolomicsworkbench.org under the submission (study id: ST001823).

## 3. Results

### 3.1. Subjects

A total of 63 horses participated in the study (AAD, *n* = 17; ABX, *n* = 15; CON, *n* = 31). The metadata of each subject (age, gender, breed, diet, antibiotic administered, indication for antibiotic, and duration of therapy) is detailed in Table 1. 

Antimicrobials given to horses in the AAD and ABX groups included ceftiofur crystalline (AAD, *n* = 4; ABX, *n* = 2), doxycycline (AAD, *n* = 4; ABX, *n* = 4), penicillin and gentamycin (AAD, *n* = 4; ABX, *n* = 4), or penicillin and gentamycin in combination with either metronidazole (AAD, *n* = 2; ABX, *n* = 2) or doxycycline (AAD, *n* = 2; ABX, *n* = 2) and trimethoprim sulfa (AAD, *n* = 1; ABX, *n* = 1). 

### 3.2. Microbiome

#### 3.2.1. Microbial Community Composition (Beta Diversity)

**Figure 1:**
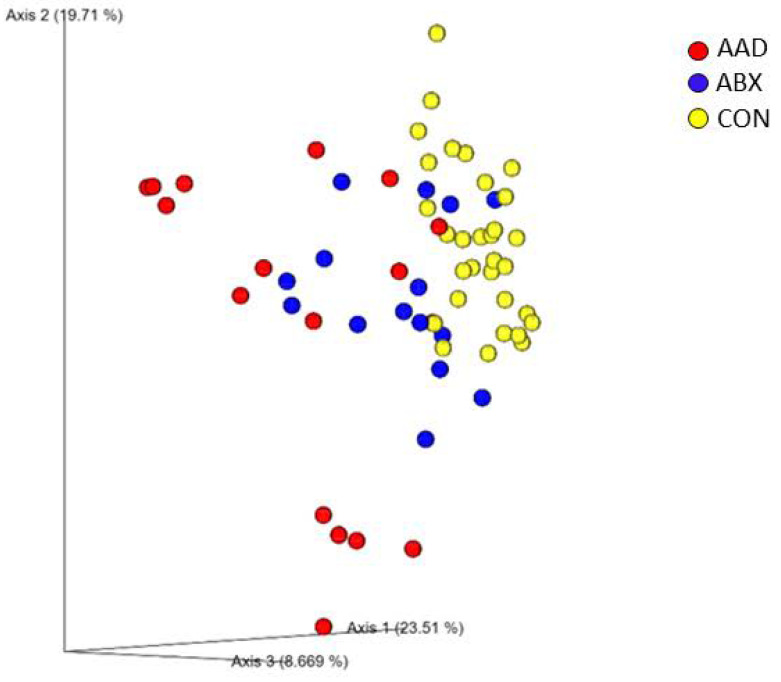
Principal coordinate analysis plot of weighted Unifrac distances horses with antimicrobial-associated diarrhea (AAD, red spheres), antibiotic control horses (ABX, blue spheres), and control horses (CON, yellow spheres). The microbial community composition of horses with AAD compared to CON horses was considered significantly different (R = 0.568, *p* = 0.001). Horses in the ABX group were different with some overlap from CON horses (R = 0.3, *p* = 0.001), and AAD and ABX horses were considered similar with high amounts of overlap (R = 0.121, *p* = 0.012).

Bacterial community composition was affected by antibiotic use, with clustering of AAD and ABX samples compared to CON horses (R = 0.391, *p* = 0.0001) on a PCoA plot of weighted Unifrac distances (Figure 1). 

AAD horses had stronger separation from CON horses (R = 0.560, *p* = 0.000) compared to ABX horses (R = 0.3, *p* = 0.0001) in the pairwise comparison. There was not a strong separation between AAD and ABX horses (R = 0.121, *p* = 0.0012) (Table 2).

#### 3.2.2. Species Richness and Evenness (Alpha Diversity)

**Figure 2:**
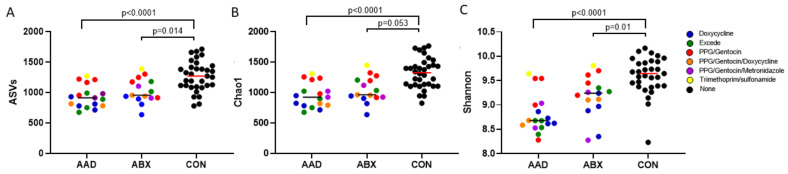
Alpha diversity metrics of horses with antimicrobial-associated diarrhea (AAD), antibiotic control horses (ABX), and control horses (CON). AAD and ABX horses show a decreased richness and evenness compared to CON horses but showed no significant difference between each other. Antibiotic use is denoted by color: doxycycline (blue), ceftiofur (green), procaine penicillin G/gentamycin (red), procaine penicillin G/gentamycin/doxycycline (orange), procaine penicillin G/gentamycin/metronidazole (purple), trimethoprim sulfonamide (yellow), and none (black). (**A**) ASV, (**B**) Chao1, (**C**) Shannon.

Metrics for richness and evenness (alpha diversity) were significantly decreased in both AAD and ABX groups compared to CON, but not to each other (Figure 2). Horses with AAD had significantly decreased amplicon sequence variants (*p* = 0.001), Chao1 (*p* = 0.0001), and Shannon (*p* = 0.0001) indices compared to CON horses. Horses with ABX had significantly less amplicon sequence variants (*p* = 0.063), Chao 1 (*p* = 0.005), and Shannon (*p* = 0.0098) metrics than CON horses. There were no significant differences in any of the three alpha diversity metrics between horses with AAD and ABX. Horses in AAD and ABX groups are colored-coded by the antibiotic protocol they received.

#### 3.2.3. Taxonomy

**Figure 3:**
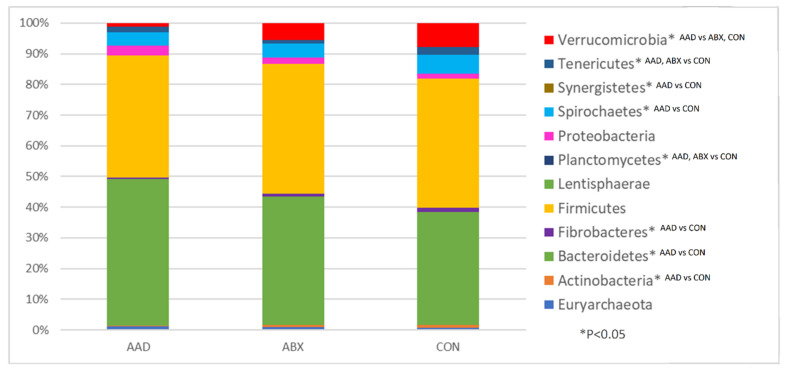
The median abundance of bacteria in the feces of horses with antimicrobial-associated diarrhea (AAD), antibiotic control horses (ABX), and control horses (CON). * denotes significant differences (*p* < 0.05) between groups.

Analysis of bacterial taxa indicated that 14 of 19 phyla (Table 3) identified had significant alterations between groups. Those with 1% or more of the total bacteria are represented on a histogram (Figure 3). 

In eight phyla, significant differences occurred between horses in the AAD and CON groups (Actinobacteria, *p* = 0.0115, q = 0.0192; Armatimonadetes, *p* = 0.0032, q = 0.008; Bacteroidetes, *p* = 0.0001, q = 0.0005; Fibrobacteres, *p* = 0.0492, q = 0.0656; SR1, *p* = 0.0001, q = 0.0005; Spirochaetes, *p* = 0.0137, q = 0.0211; Synergistetes, *p* = 0.0114, q = 0.1912; TM7, *p* = 0.0075, q = 0.0167. In all of these phyla except for Bacteroidetes, there was a decrease in the bacterial abundance of the AAD group compared to the CON group. In three phyla, there were differences between CON horses and both groups of horses given antimicrobials, AAD and ABX: Elusimicrobia, *p* = 0.001, q = 0.005; Planctomycetes, *p* = 0.0002, q = 0.0008; and SR1, *p* = 0.0001, q = 0.0005. In three phyla, there were differences between ABX and CON horses (Fusobacteria, *p* = 0.0214, q = 0.0306; Tenericutes, *p* = 0.0004, q = 0.0013; WPS-2, *p* = 0.0109, q = 0.01917). In these three phyla, the abundance of bacteria was decreased in the antibiotic groups, AAD and ABX. Only in one phylum, Verrucomicrobia, were there changes between horses with diarrhea (AAD) and with normal feces and health status (ABX and CON) (*p* = 0.0001, q = 0.0005). 

AAD horses had less of these Actinobacteria than CON horses (*p* = 0.0115, q = 0.0192). These changes were attributable to the class Actinobacter, order Actinomycetales, and the families Norciadiaceae (*p* = 0.0171, q = 0.0519), Bifidobacteriaceae (0.0082, q = 0.0275), and Coriobacteriaceae (*p* = 0.0024, q = 0.0107), all of which had greater abundance in CON than AAD horses.

AAD horses had significantly less Armatimonadetes than CON horses (*p* = 0.0032, q = 0.008). In the class SJA-176, order RB046, an unknown family (*p* = 0.0032, q = 0.0137) had significantly greater abundance in CON than AAD horses. 

AAD horses had significantly more Bacteroidetes than CON horses (*p* = 0.0001, q = 0.0005). In the class Bacteroidia and order Bacteroidales, the families Bacteroidaceae (*p* = 0.001, q = 0.0012), Porphyromonadadeae (*p* = 0.0035, q = 0.014), and Prevotellaceae (*p* = 0.0313, q = 0.0754) all had greater abundance in AAD than CON horses.

CON horses had greater abundance of Elusimicrobia than AAD or ABX horses (*p* = 0.001, q = 0.005). For the class Elusimicrobia and order Elusicmicrobiales, the family Elusimicrobiaceae (*p* = 0.0034, q = 0.0137) and unknown order, unknown family (*p* = 0.002, q = 0.0016), were higher in CON horses than in AAD horses. 

Within the phyla Fibrobacteres, CON horses had a greater abundance than AAD horses (*p* = 0.0492, q = 0.0656). The family Fibrobacteraceae (*p* = 0.0492, q = 0.1101) in the class Fibrobacteria and order Fibrobacterales had a greater abundance in CON horses compared to AAD horses. 

In the phyla Firmicutes, the class Bacilli, order Lactobacillales, the families Aerococcaceae (*p* = 0.0065, q = 0.0235) and Enterococcaceae (*p* = 0.0177, q = 0.0520) had greater abundance in CON horses compared to AAD horses. In the class Clostridia and order Clostridiales, an unknown bacteria (*p* = 0.0039, q = 0.0147), unknown family (*p* = 0.0245, q = 0.0645), Clostridiaceae (*p* = 0.002, q = 0.0016), EtOH8 (*p* = 0.0001, q = 0.1112), Eubacteriaceae (*p* = 0.001, q = 0.0012), Peptostreptococcaceae (*p* = 0.0001, q = 0.0012), and Mogibacteriaceae (*p* = 0.0002, q = 0.0015) had greater abundance in CON than AAD horses. 

ABX horses had increased amounts of Fusobacteria compared to CON horses (*p* = 0.0214, q = 0.0306). The family Fusobacteriaceae (*p* = 0.0214, q = 0.0591) from the class Fusobacteria and order Fusobacteriales was increased in AAD compared to CON horses. 

Within the Phylum Lentisphaerae class (Lentisphaeria), order Z20, the family R4-45B (*p* = 0.0247, q = 0.645) was greater in CON horses compared to AAD horses. 

Horses on antimicrobials (AAD and ABX) had significantly less Planctomycetes than CON horses (*p* = 0.0002, q = 0.0008). The family Pirellulaceae (*p* = 0.001, q = 0.0012) from the class Planctomycetia and order Pirellulales was higher in AAD and ABX horses than CON horses. Within the class vadinHA49 and order PeHg47, an unknown family (*p* = 0.0008, q = 0.0044) was more abundant in CON compared to AAD horses. 

Within the phylum Proteobacteria, class Alphaproteobacteria, an unknown order and class (*p* = 0.008, q = 0.0044) was more abundant in CON than AAD horses. In the order RF32, an unknown family (*p* = 0.0006, q = 0.004) was more abundant in CON than AAD horses. Within the order Rickettsiales, an unknown family (*p* = 0.0387, q = 0.0887) was more abundant in CON than AAD horses. In the class Deltaproeobacteria and order Desulfovibrionales, the family Desulfovibrionaceae (*p* = 0.0191, q = 0.0544) was more abundant in AAD horses compared to CON horses. However, there was a greater abundance of an unknown family from the order GMD14H09 (*p* = 0.0007, q = 0.0044) and an unknown family from the order Myxococcales, (*p* = 0.03, q = 0.0754). Within the class Gammaproteobacteria and the order Enterobaceriales, the family Enterobacteriaceae (*p* = 0.0016, q = 0.0084) and the family Moraxellaceae from the order Pseudomonadales (*p* = 0.0001, q = 0.0012) were more abundant in AAD horses than CON horses. 

Within the phylum Spirochaetes, class Spirochaetes and order Spirochaetales, the family Spirochaetaceae (*p* = 0.0143, q = 0.0448) was increased in CON compared to AAD horses. The family Synergistaceae (*p* = 0.0074, q = 0.0258) within the class Synergistia and order Synergistales was increased in AAD compared to CON horses. 

CON horses had increased amounts of SR1 compared to AAD and ABX horses (*p* = 0.0001, q = 0.0005). 

In the phyla TM7, class TM7, order CW040, the family F16 (*p* = 0.0074, q = 0.0258) was increased in CON compared to AAD horses. 

CON horse had significantly more Tenericutes than ABX horses (*p* = 0.0004, q = 0.0013). The family Anaeroplasmataceae (*p* = 0.0306, q = 0.0754) from the class Mollicutes and order Anaseroplasmatales was increased in AAD horses compared to CON horses, whereas the family Mycoplasmataceae (*p* = 0.0354, q = 0.0832) from the order Mycoplasmatales and an unknown family (*p* = 0.0001, q = 0.0012) from the order RF39 were decreased in AAD compared to CON horses.

Horses with diarrhea (AAD) had significantly less Verrucomicrobia compared to both subsets of healthy horses (ABX and CON) (*p* = 0.0001, q = 0.0005). Three families, unknown (*p* = 0.0018, q = 0.0089), RPF12 (*p* = 0.0001, q = 0.0012), and WCHB1-25 (*p* = 0.0022, q = 0.0103) from the class Verruco-5 and order WCHB1-41 were decreased in AAD compared to CON horses. 

CON horses had significant increased abundances of WPS2 compared to ABX horses (*p* = 0.0109, q = 0.01917). An unknown class, order, and family (*p* = 0.0109, q = 0.0353) from the phyla WPS-2 accounted for this change. 

Tables S1 and S2 contain the median abundance of each group at the family and species levels.

#### 3.2.4. Linear Discriminant Analysis Effect Size (LEfSe)

**Figure 4:**
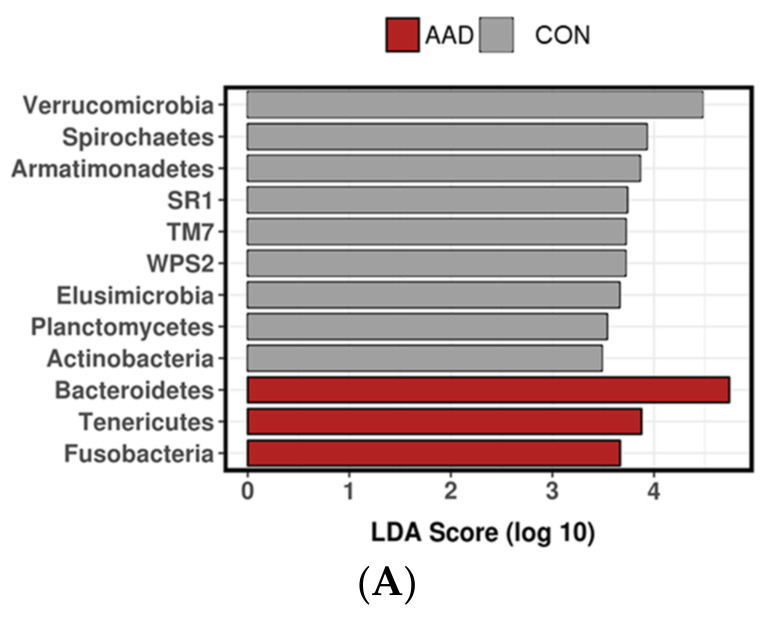

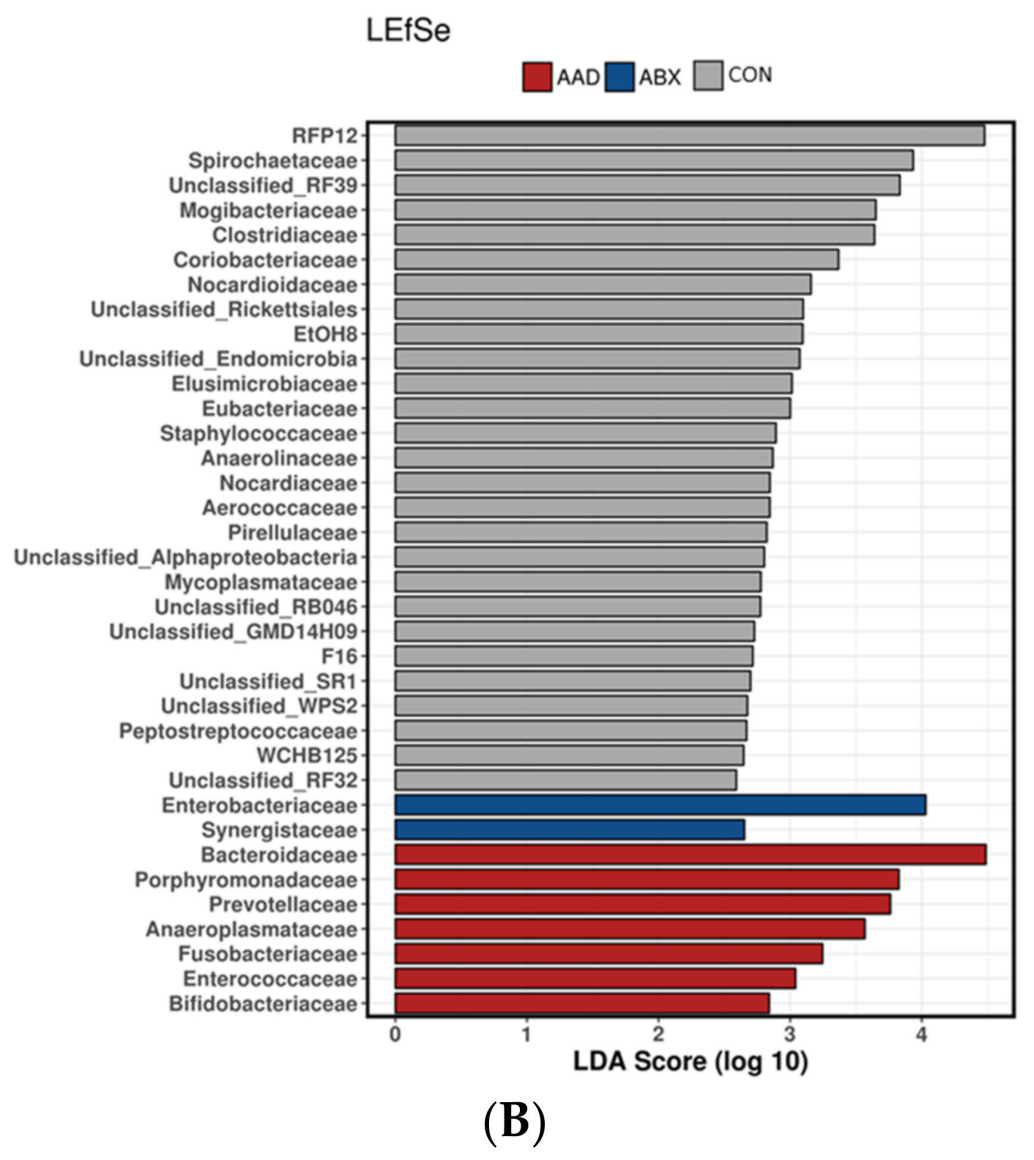
Linear discriminant analysis effect size (LEfSe) analysis at the (**A**) phylum and (**B**) family levels in horses with antimicrobial-associated diarrhea (AAD, red bars), antibiotic control horses (ABX, blue bars), and control horses (CON, gray bars).

The linear discriminant analysis effect size (LEfSe) at the phylum level indicated Bacteroidetes, Fusobacteria, and Tenericutes were differentially expressed by horses with AAD, whereas Actinobacteria, Armatimonadetes, Elusimicrobia, SR1, Spirochaetes, and TM7 were differentially expressed by CON horses. The results of the LEfSe analysis at the phylum and family levels are displayed in Figure 4A,B.

**Figure 5:**
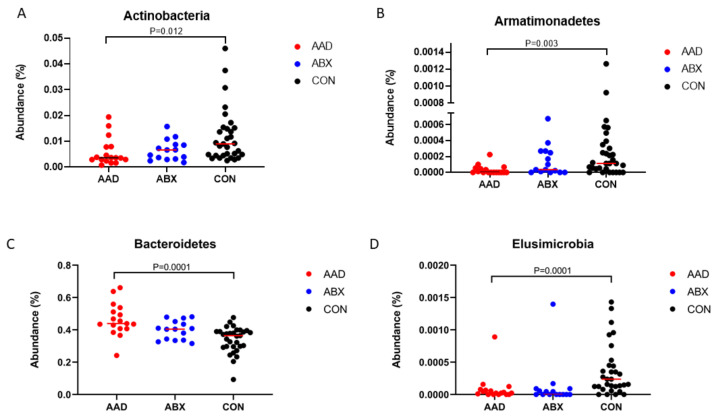

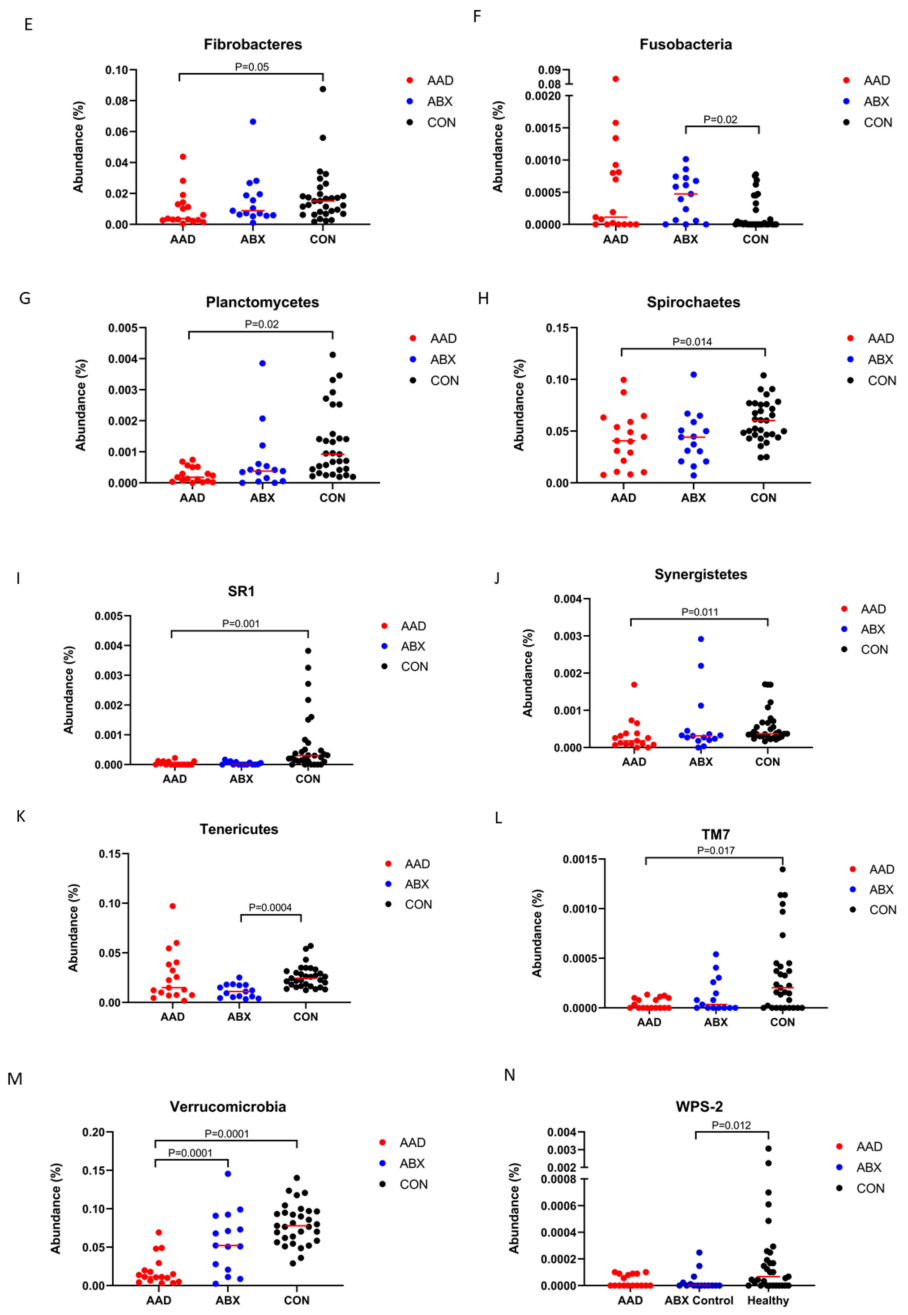
Scatter plots of the median abundance of phyla found to be significantly different after linear discriminant analysis effect size (LEfSe) analysis. Horses with antimicrobial-associated diarrhea (AAD) are represented by red spheres, antibiotic control horses (ABX) by blue spheres, and non-antibiotic control horses (CON) by black spheres. (**A**) Actinobacteria, (**B**) Armatimonadets, (**C**) Bacteroidetes, (**D**) Elusimicrobia, (**E**) Fibrobacteria, (**F**) Fusobacteria, (**G**) Plactomycetes, (**H**) Spirochaetes, (**I**) SR1, (**J**) Synergistes, (**K**)Tenericutes, (**L**) TM7, (**M**) Verrucomicrobia, (**N**) WPS-2.

The results of the LEfSe analysis at the phylum, family, and species levels are displayed in Supplementary Tables S3–S5. Scatter plots of the phyla found to be significantly different are displayed in Figure 5.

### 3.3. Metabolomics

**Figure 6:**
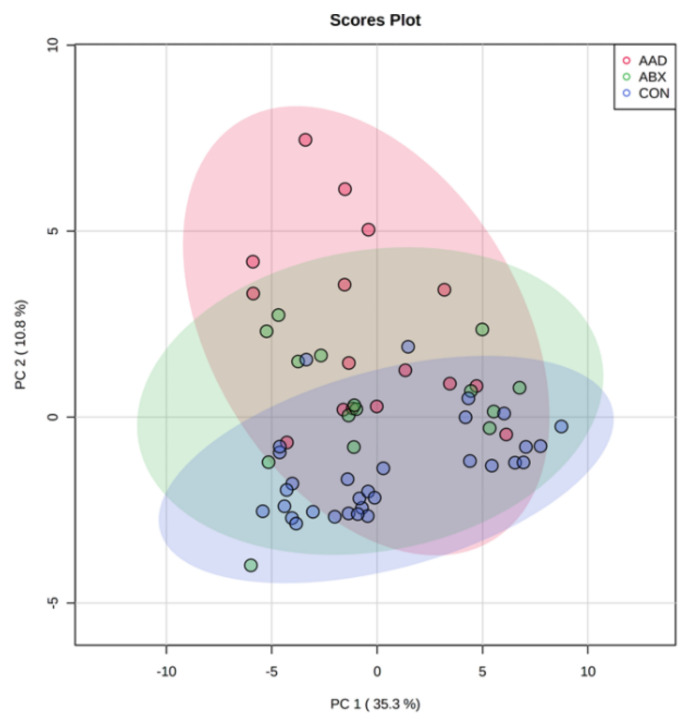
Principal component analysis plot (PCA) of horses with antimicrobial-associated diarrhea (AAD, red spheres), antibiotic control horses (ABX, green spheres), and control horses (CON, blue spheres) with shaded areas representing 95% confidence intervals.

Using an untargeted approach, a total of 1398 unique metabolites were detected, including 127 named metabolites. Metabolites were analyzed by group (AAD vs. ABX vs. CON) using PCA score plots, heat maps, and multivariate analysis. PCA plots indicated clustering of samples based upon group, with separation of AAD horses from CON and ABX horses (Figure 6).

**Figure 7:**
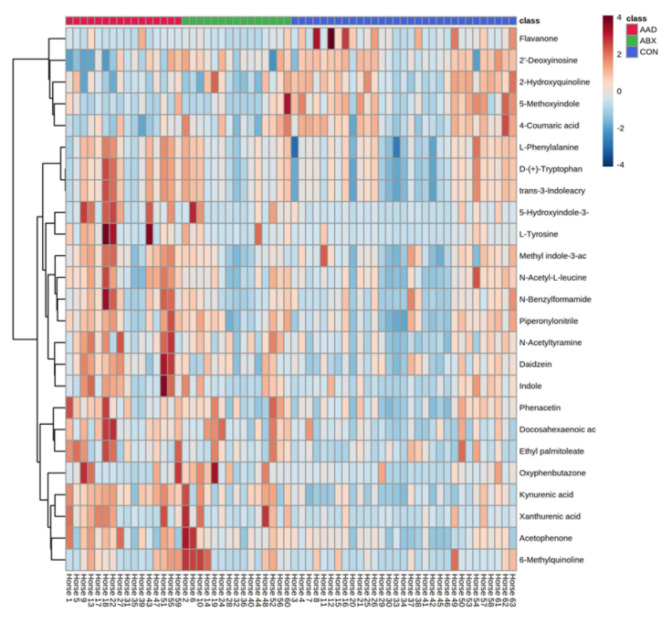
Heatmap of the 25 most abundant metabolites found in fecal samples. Each column represents an individual horse, sorted by group (antimicrobial-associated diarrhea or AAD, red; antibiotic control horses or ABX, green; control horses or CON, blue), and each row represents a metabolite. The color of each box indicates an increase (red) or decrease (blue) in metabolite concentration.

The concentration of the 25 most abundant metabolites by individual horse and group is displayed in a heatmap in Figure 7.

Seven metabolites were found to be significantly different following analysis with an ANOVA and a Tukey’s post-test (Table 4). Two of these metabolites, N-acetyltryramine and 5-hyroxyindole-3-acetic acid, were different between AAD and CON horses. Three metabolites, kynuremic acid, docosashexaenoic acid, and xanthurenic acid, demonstrated separation between horses on antibiotics versus non-antibiotic controls. Only two metabolites, daidzein and L-tyrosine, differentiated AAD horses from either control group. 

**Figure 8:**
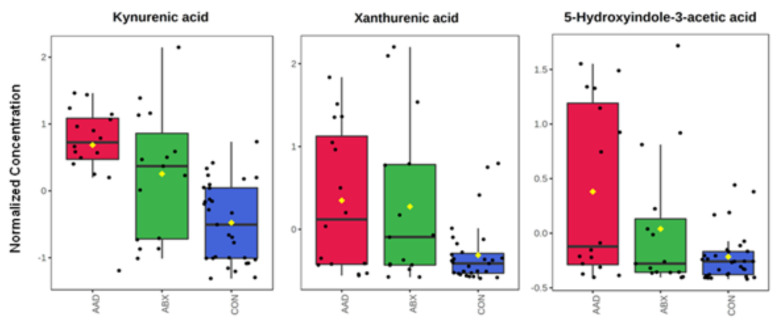
Metabolites associated with tryptophan metabolism in the feces of horses with antimicrobial-associated diarrhea (AAD), antibiotic control (ABX), and healthy control horses (CON).

Scatter plots of tryptophan metabolites are found in Figure 8.

**Figure 9:**
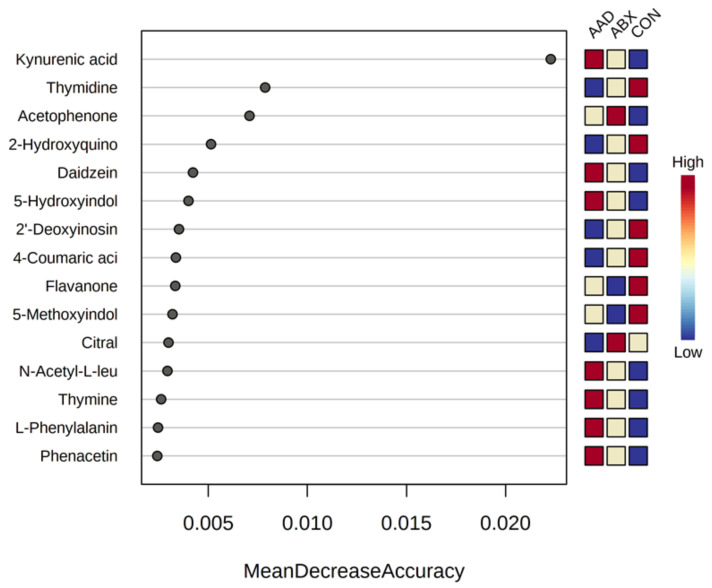
Variable importance plot for random forest evaluation of metabolites in horses with antimicrobial-associated diarrhea (AAD), antibiotic control (ABX), and healthy control horses (CON). Metabolites with the highest mean decrease accuracy (MDA) contributed the most to classification of horses as AAD, ABX, or CON.

A variable importance plot of random forest analysis found four metabolites contributed differentially to membership in AAD (kynurenic acid), ABX (thymidine), and CON horses (acetophenone) (Figure 9).

## 4. Discussion

Diarrhea is a recognized adverse effect of antibiotic therapy in horses, with clinical symptoms that range from mild and self-limiting to severe and life-threatening [29]. While the exact mechanism by which antimicrobials induce diarrhea in horses is speculative, research from human patients with AAD implicates changes in the microbial communities of the hindgut with subsequent functional effects on gut metabolism. In humans, antibiotics deplete the commensal bacteria of the gut leading to the overgrowth of enteric pathogens [30,31,32]. In addition, antibiotics reduce the concentration of short-chain fatty acids in the intestinal lumen, resulting in the accumulation of carbohydrates and bile acids leading to osmotic diarrhea [31,32]. The resulting dysbiosis caused by antibiotics may be long lasting [30,31,33]. Preliminary studies confirm that antibiotics diminish the bacterial diversity of the horse’s fecal microbiome and induce metabolic changes even when diarrhea does not occur [16,19,21,22]. To date, it is not clearly understood why some horses develop AAD or experience severe illness compared to others. Presumably, horses with ADD have greater disturbances of the bacterial communicates of the hindgut and subsequent metabolic changes that result in more severe clinical symptoms. 

This study compared the fecal microbiome and metabolome in horses that developed AAD to an antibiotic (ABX) and a non-antibiotic (CON) control group. In an effort to limit sources of variation in the study, the authors matched subjects for two factors that directly impacted the fecal microbiome, diet and antibiotic use. Horses on antimicrobial therapy (AAD and ABX) were matched by antibiotic agent, dose, route of administration, and days of exposure. Horses from all three groups (AAD, ABX, and CON) were matched by diet. As horses in the AAD and ABX groups came from the same hospital population, they were fed the same feed and were housed under identical environmental conditions. In order to match their diet with CON horses that were not hospitalized, a dietary scale was used to categorize the intake of forages and concentrates. Ideally, horses would be matched for other variables, such as age [20,34], breed [35], and gender. While these factors can have a minor influence on the fecal microbiome, practicality necessitated that the authors control for factors with the greatest influence, antimicrobial agent [21], days of antimicrobial exposure [10,12,20], and diet [23]. 

This study did not stratify horses by individual antibiotic agent or mechanism of action. Horses on six different antimicrobial protocols were included, some of which had distinct mechanisms of action and bacterial spectrums, which could have differing effects on the microbiome and metabolome. Studies in humans have indicated that antimicrobials can induce unique effects on the composition of the microbiome [36], with the responses of macrolides [37], clarithromycin [38], vancomycin [39], clindamycin [40], and ciprofloxacin [41] described. In humans, the antibiotics generally induce increases in Bacteroidetes and Proteobacteria, while Firmicutes and Actinobacteria are suppressed [37]. The horse’s microbiome appears to have a similar response to antibiotics [6,15,42], but only a few studies have utilized 16S sequencing to characterize these changes [17,21,23]. These studies have demonstrated that antibiotic agents, regardless of their mechanism of action, appear to dramatically diminish the diversity (including species richness and evenness) of the equine fecal microbiome. This result is similar to other studies in which antimicrobials were administered to healthy horses or to clinical patients that subsequently developed diarrhea [16,18,19,20,21,22,23,24,42,43,44]. While AAD horses had the lowest median score for each alpha diversity metric, they were not statistically different from the antibiotic control horses. Reduction in diversity of the microbiome is a persistent feature of both antibiotic use and colitis and may be associated with common metabolic pathways. Ideally, future studies will be able to stratify the effects on the microbiome and metabolome by individual antimicrobial agents.

While alpha diversity metrics were equally augmented in AAD and ABX groups, horses with AAD had the greatest differences in microbial community composition when compared to CON horses and minor changes when compared to ABX horses. This is reflected on the PCA plot and supported by the univariable analysis. The fecal microbiome of AAD horses had significant differences in eight phyla compared to CON horses. AAD horses showed an increased abundance of Bacteroidetes and decreased abundances of Actinobacteria, Armatimonadetes, Fibrobacteres, SR1, Spirochaetes, Synergistetes, and TM7 compared to CON horses. Firmicutes, an important phylum that constitutes a majority of the gut bacteria, was decreased in AAD horses compared to ABX and CON horses, but this result did not obtain statistical significance. Non-antibiotic-treated control horses showed an increased abundance of Elusimicrobia, Planctomycetes, and SR1 compared to AAD and ABX horses. Antimicrobial use appears to deplete the commensal bacteria of the equine microbiome, which may allow for expansion of other phyla, such as Bacteroidetes and Fusobacteria. 

While the abundance of many phyla was significantly different between AAD and CON horses, only a select number of taxa distinguished horses that developed colitis (AAD) from those that maintained normal fecal character and health status during antibiotic therapy (ABX). This included Bifidobacteriaceae, Bacteroidaceae, Prevotellaceae, and Enterococcaceae, which were increased in AAD horses compared to ABX and CON horses, and Mogibacteriaceae, which was decreased in AAD horses compared to ABX and CON horses. One of the most dramatic changes occurred in the phylum Verrucomicrobia, where the abundances of class Verruco-5, order WCHB1-41, and family RPF-12 were markedly decreased compared to ABX and CON horses. Generally, the bacteria that were decreased in AAD are considered important mutualist or commensal bacteria, whereas the bacteria that were increased are found elevated in reports of horses with gastrointestinal disease, such as colic or colitis [12,16,18,19,21,22,23,24]. In all cases, the same trends for these phyla also occurred in the ABX group but to a lesser extent, which did not result in statistical significance between AAD and ABX horses. 

The selective decrease in Verrucomicrobia in AAD horses suggests a mechanism by which antimicrobials agents may induce equine colitis. The mucus layer that separates the epithelial cells from the luminal content of the gut plays an important role in barrier function. Verrucomicrobia maintains the mucus layer at the epithelial cell–luminal interface of the large intestine by utilizing the carbon and nitrogen content of the mucus layer as an energy source. This promotes constant turnover, which results in a healthy mucin layer at the epithelial–lumen interface [45]. Compromise of this mucin layer may allow overgrowth of pathogenic species of bacteria, such as *Salmonella* or *Clostridia*, and promote inflammation. Verrucomicrobia was dramatically decreased in horses with AAD compared to horses on antibiotics and non-antibiotic control horses. CON and ABX horses had approximately seven and five times the amount of RFP12 compared to horses with AAD. This same effect has been noted in other studies involving horses on antibiotics and those that developed AAD [17,21,23,24,25], which may indicate the usefulness of this bacteria as a future biomarker for AAD or as a potential target for therapeutic intervention.

As the gut microbiome strongly influences the gut metabolome, dysbiosis alters host metabolism [46]. Metabolomic analysis of humans and dogs has detected changes in the metabolite profiles of individuals with colitis compared to healthy subjects and has provided information regarding the specific pathways affected [47,48,49,50]. Metabolomic analysis is relatively new in equine research but has provided insight into the metabolic pathways in equine metabolic diseases [51], which may share some overlap with colitis. In this study, the univariable analysis identified seven metabolites that were significantly different across groups. Three of the metabolites, 5-hyroxyindole-3-acetic acid and kynurenic and xanthurenic acids, originate from tryptophan metabolism [50] and showed differences between AAD and/or ABX horses and CON horses. Tryptophan, an essential amino acid, is important for epithelial cell barrier function in the gut. Tryptophan is catabolized by the gastrointestinal microbiota into three main pathways, indole, serotonin, and kynurenine pathways [52]. Kynurenic and xanthurenic acid are products of the kynurenine pathway, whereas 5-hyroxyindole-3-acetic acid is part of the indole pathway. In general, theses tryptophan metabolites bind to an aryl hydrocarbon receptor (AHR) and enhance the epithelial barrier function, stimulate gastrointestinal motility, secrete gut hormones and exert an anti-inflammatory effect, and modulate the gut microbial composition [52]. Similar to humans and dogs, these metabolites are increased in the feces of horses with AAD and/or ABX. This may reflect a loss of active metabolite in the gastrointestinal tract and, consequently, result in poor barrier function and subsequent colonic inflammation. 

Two other metabolites, daidzein and L-tyrosine, were increased in horses with AAD from ABX and CON horses. Daidzein is an isoflavone, initially isolated from the urine of pregnant horses and used as hormonal replacement therapy in post-menopausal women [53,54]. Isoflavone phytoestrogens are structurally similar to natural estrogens and found in leguminous plants commonly used in hay or pasture grasses [53]. They are absorbed in the small intestine, transported to the liver to undergo conjugation, and released back to the intestine in bile [55]. The colonic microbiota converts isoflavones into daidzein. Isoflavones and their metabolites have an inhibitory effect on glycolysis and promote lipid metabolism, which may be altered in horses with colitis [53,54]. 

L-tyrosine is a non-essential amino acid that is a precursor to the catecholamine neurotransmitters dopamine, norepinephrine, and epinephrine. It also is a precursor to tyramine. Both tyrosine and tyramine are increased in horses with AAD compared to CON, similar to dogs and humans with inflammatory bowel disease [48,56]. These metabolites may have a role in the increased gut motility associated with colitis.

## 5. Conclusions

In conclusion, antibiotics appear to deplete the commensal bacteria of the horse’s hindgut and alter specific metabolic pathways. Horses that develop diarrhea secondary to antibiotic use have marked decreases in the phylum Verrucomicrobia and altered metabolite profiles compared to ABX and CON horses. These changes in amino acid and lipid metabolism may affect epithelial barrier function, motility, and energy metabolism in horses that develop AAD. 

## Figures and Tables

**Table 1:** Horse id, breed, sex, age, diet, group, antibiotic, duration of therapy, and indication for antibiotic. Color blocking indicates matching of antimicrobial-associated diarrhea (AAD), antibiotic control (ABX), and non-antibiotic control (CON) horses. The alternating background color indicates groupings of animals, i.e., matching of an AAD, ABX and CON horse group.

Horse ID	Breed	Sex	Age	Diet Type	Group	Antibiotic	Days on ABX	Reason for ABX
Horse 104	Quarter Horse	Mare	1	D	AAD	Doxycycline	4	Acute facial fracture
Horse 147	Quarter Horse	Mare	1	D	ABX	Doxycycline	2	Over-riding dorsal spinous processes
Horse 58	Tennessee Walker	Mare	3	D	CON	None	0	Healthy control
Horse 52	Quarter Horse	Mare	2	D	CON	None	0	Healthy control
Horse 148	Quarter Horse	Gelding	9	C	AAD	Doxycycline	7	Pemanent tracheostomy
Horse 149	Quarter Horse	Gelding	12	C	ABX	Doxycycline	8	Wound revision
Horse 150	Mix	Gelding	8	C	CON	None	0	Healthy control
Horse 45	Warmblood	Gelding	7	C	CON	None	0	Healthy control
Horse 151	Quarter Horse	Gelding	6	B	AAD	Doxycycline	5	Tie-forward
Horse 152	Quarter Horse	Gelding	15	B	ABX	Doxycycline	7	Tenoscopy
Horse 36	Quarter Horse	Gelding	12	B	CON	None	0	Healthy control
Horse 48	Mix	Gelding	7	B	CON	None	0	Healthy control
Horse 108	Quarter Horse	Stallion	1	C	AAD	Excede	10	Pneumonia
Horse 153	Miniature Donkey	Stallion	1	C	AAD	Excede	7	Pneumonia
Horse 154	Thoroughbred	Gelding	12	C	ABX	Excede	5	Dental/sinus disease
Horse 42	Warmblood	Gelding	6	C	CON	None	0	Healthy control
Horse 46	Quarter Horse	Gelding	2	C	CON	None	0	Healthy control
Horse 102	Quarter Horse	Gelding	9	D	AAD	Excede	3	Pneumonia
Horse 155	Paint	Stallion	1	D	AAD	Excede	2	Pneumonia
Horse 156	Quarter Horse	Mare	19	D	ABX	Excede	4	Oocyte aspiration
Horse 61	Tennessee Walking Horse	Stallion	8	D	CON	None	0	Healthy control
Horse 63	Draft cross	Gelding	9	D	CON	None	0	Healthy control
Horse 111	Paint	Mare	11	D	AAD	Penicillin, Gentocin	4	Mass removal
Horse 159	Quarter Horse	Mare	8	D	ABX	Penicillin, Gentocin	3	Fetlock arthrodesis
Horse 55	Warmblood	Mare	8	D	CON	None	0	Healthy control
Horse 60	Quarter Horse	Mare	14	D	CON	None	0	Healthy control
Horse 106	Quarter Horse	Stallion	5	C	AAD	Penicillin, Gentocin	4	Acute facial fracture
Horse 161	Warmblood	Mare	12	C	ABX	Penicillin, Gentocin	4	Pastern arthrodesis
Horse 162	Quarter Horse	Stallion	3	C	CON	None	0	Healthy control
Horse 163	Quarter Horse	Stallion	6	C	CON	None	0	Healthy control
Horse 164	Warmblood	Gelding	1	C	AAD	Penicillin, Gentocin	1	Cryptorchid
Horse 165	Quarter Horse	Gelding	2	C	ABX	Penicillin, Gentocin	1	Stifle arthroscopy
Horse 166	Quarter Horse	Gelding	1	C	CON	None	0	Healthy control
Horse 28	Warmblood	Gelding	2	C	CON	None	0	Healthy control
Horse 103	Quarter Horse	Stallion	1	D	AAD	Penicillin, Gentocin	3	Inferior check ligament desmotomy
Horse167	Quarter Horse	Stallion	1	D	ABX	Penicillin, Gentocin	3	Cryptorchid
Horse 61	Quarter Horse	Stallion	3	D	CON	None	0	Healthy control
Horse 168	Draft	Stallion	1	D	CON	None	0	Healthy control
Horse 116	Mixed	Gelding	1	B	AAD	Penicillin, Gentocin, Doxycycline	7	Pneumonia
Horse 169	Saddlebred	Stallion	1	B	ABX	Penicillin, Gentocin, Doxycycline	7	Radial fracture
Horse 170	Quarter Horse	Gelding	7	B	CON	None	0	Healthy control
Horse 171	Tennessee Walker	Stallion	6	B	CON	None	0	Healthy control
Horse 105	Warmblood	Gelding	12	C	AAD	Penicillin, Gentocin, Doxycycline	7	Fasciotomy/neurectomy
Horse 172	Paint	Gelding	10	C	ABX	Penicillin, Gentocin, Doxycycline	7	Ulnar fracture
Horse 40	Quarter Horse	Gelding	8	C	CON	None	0	Healthy control
Horse 178	Warmblood	Gelding	16	C	CON	None	0	Healthy control
Horse 113	Quarter Horse	Mare	12	B	AAD	Penicillin, Gentocin, Metronidazole	3	Pneumonia
Horse 173	Warmblod	Gelding	19	B	ABX	Penicillin, Gentocin, Metronidazole	4	Pneumonia
Horse 30	Quarter Horse	Mare	13	B	CON	None	0	Healthy control
Horse 25	Quarter Horse	Mare	12	B	CON	None	0	Healthy control
Horse 107	Thoroughbred	Mare	25	D	AAD	Penicillin, Gentocin, Metronidazole	4	Pneumonia
Horse 174	Criollo	Mare	19	D	ABX	Penicillin, Gentocin, Metronidazole	4	Acute facial trauma
Horse 175	Quarter Horse	Mare	24	D	CON	None	0	Healthy control
Horse 59	Thoroughbred	Mare	12	D	CON	None	0	Healthy control
Horse 176	Miniature Horse	Gelding	26	D	AAD	TMS	7	Dental/sinus disease
Horse 177	Miniature Horse	Gelding	23	D	ABX	TMS	14	Dental/sinus disease
Horse 62	Appoloosa	Gelding	21	D	CON	None	0	Healthy control
Horse 63	Thoroughbred	Gelding	24	D	CON	None	0	Healthy control
Horse 56	Quarter Horse	Gelding	19	D	CON	None	0	Healthy control

**Table 2:** Unweighted and weighted ANOSIM values for horses with antimicrobial-associated diarrhea (AAD), antibiotic control horses (ABX), and control horses (CON).

Group	Unweighted		Weighted	
	R	*p*-Value	R	*p*-Value
Overall	0.398	0.001	0.391	0.001
AAD vs. ABX	0.063	0.078	0.121	0.0012
AAD vs. CON	0.547	0.001	0.568	0.001
ABX vs. CON	0.37	0.001	0.3	0.001

**Table 3:** The median abundance of phyla in horses with antimicrobial-associated diarrhea (AAD), antibiotic control (ABX), and control horses (CON). Groups with differing superscripts (a or b) denote significant differences among groups.

Bacterial Phyla	AAD		ABX		CON		AAD vs. ABX vs. CON	
	Median	Range	Median	Range	Median	Range	*p*-Value	Q-Value
Euryarchaeota	0.98	0.1–2.12	0.84	0.07–1.98	0.78	0.02–1.43	0.4535	0.4535
Unknown	0	0–0.04	0.01	0–0.03	0	0–0.03	0.25	0.2632
Actinobacteria	0.36 ^a^	0.08–1.94	0.66 ^a,b^	0.18–1.57	0.9 ^b^	0.25–4.6	0.0115	0.0192
Armatimonadetes	0 ^a^	0–0.02	0 ^a,b^	0–0.07	0.01 ^b^	0–0.13	0.0032	0.008
Bacteroidetes	43.88 ^a^	24.16–66.11	40.43 ^a,b^	31.59–48.07	36.46 ^b^	9.29–47.6	0.0001	0.0005
Elusimicrobia	0 ^a^	0–0.09	0 ^a^	0–0.14	0.02 ^b^	0–0.14	0.0001	0.0005
Fibrobacteres	0.38 ^a^	0.06–4.38	0.89 ^a,b^	0.1–6.65	1.51 ^b^	0.18–8.75	0.0492	0.0656
Firmicutes	36.45	21.78–54.83	40.8	28.59–58.12	41.68	29.81–68.78	0.2058	0.2287
Fusobacteria	0.01 ^a,b^	0–8.36	0.05 ^a^	0–0.1	0 ^b^	0–0.08	0.0214	0.0306
Lentisphaerae	0.02	0–0.37	0.07	0.01–0.31	0.05	0–0.61	0.1373	0.1615
Planctomycetes	0.02 ^a^	0–0.07	0.04 ^a^	0–0.39	0.09 ^b^	0.02–0.41	0.0002	0.0008
Proteobacteria	2.93	0.88–14.32	1.97	0.28–25.99	1.59	0.37–10	0.0695	0.0860
SR1	0 ^a^	0–0.02	0 ^a^	0–0.02	0.03 ^b^	0–0.38	0.0001	0.0005
Spirochaetes	4.07 ^a^	0.77–9.96	4.41 ^a,b^	0.72–10.46	6.03 ^b^	2.44–10.39	0.0137	0.0211
Synergistetes	0.02 ^a^	0–0.17	0.03 ^a,b^	0–0.29	0.04 ^b^	0.02–0.17	0.0114	0.0192
TM7	0 ^a^	0–0.01	0 ^a,b^	0–0.05	0.02 ^b^	0–0.14	0.0075	0.01667
Tenericutes	1.5 ^a,b^	0.18–9.72	1.11 ^a^	0.34–2.52	2.44 ^b^	1.24–5.7	0.0004	0.00133
Verrucomicrobia	1.15 ^a^	0.31–6.93	5.23 ^b^	0.24–14.55	7.78 ^b^	2.9–14.02	0.0001	0.0005
WPS-2	0 ^a,b^	0–0.01	0 ^a^	0–0.02	0.01 ^b^	0–0.31	0.0109	0.01917

**Table 4:** Fecal metabolites that were significantly different between horses with antimicrobial-associated diarrhea (AAD), antibiotic control (ABX), and control horses (CON).

Group	Metabolite	*p*-Value	FDR	Perturbation to CON	Source	KEGG Pathway	Specific Pathway
AAD vs. CON	N-acetyltyramine	0.0017	0.0262	Increased in AAD	Endogenous	Amino acid metabolism	Precursor for L-tyrosine
	5-hydroxyindole-3-acetic acid	0.0022	0.0290	Increase in AAD	Gut microbiota	Amino acid metabolism	Tyrptophan, indole pathway
AAD, ABX vs. CON	Kynurenic acid	0.0000	0.0004	Increased in AAD, ABX	Gut microbiota	Amino acid metabolism	Tryptophan, kynurenine pathway
	Docosahexaenoic acid	0.0015	0.0262	Increased in AAD, ABX	Dietary	Lipid metabolism	Omega-3 essential fatty acid
	Xanthurenic acid	0.0033	0.0348	Increased in AAD, ABX	Gut microbiota	Amino acid metabolism	Tryptophan, kynurenine pathway
AAD vs. ABX, CON	Daidzein	0.0009	0.0215	Increased in AAD	Gut microbiota	Biosynthesis of secondary metabolites	Isoflavone metabolite, estrogenic compound
	L-tyrosine	0.0030	0.0348	Increase in AAD	Dietary	Amino acid metabolism	Precursors for dopamine, norepinephrine, and epinephrine

## Data Availability

Publicly available datasets were utilized in this study. The data can be found here: metabolomicsworkbench.org, (ST001823); NCBI Sequence Read Archive, bioproject number PRJNA728793; KEGG Pathway Database at www.genome.jp/kegg/pathway, accessed on 22 January 2021.

## Supplementary Materials

The following are available online at https://www.mdpi.com/article/10.3390/ani11061807/s1, Table S1: Median abundance of taxa at the family level for horses with antimicrobial-associated diarrhea (AAD), antibiotic control horses (ABX), and non-hospitalized control horses (CON); Table S2: Median abundance of taxa at the species level for horses with antimicrobial-associated diarrhea (AAD), antibiotic control horses (ABX), and control horses (CON); Table S3: The results of LEfSe analysis at the phylum level; Table S4: The results of LEfSe analysis at the family level; Table S5: The results of LEfSe analysis at the species level.
